# Supplementary material for: Analysis of PPARγ Signaling Activity in Psoriasis
Source: Int J Mol Sci. 2021 Aug 10;22(16):8603. doi: 10.3390/ijms22168603 (PMC8395241; doi:10.3390/ijms22168603)
Supplement: Supplementary file 1 [file ijms-22-08603-s001.zip › Supplemental materials_Analysis of PPARg signaling activity in psoriasis/Pathway models/Models images and html files/Model of PPARG signaling in psoriasis/1759.html]

CASP1


# Protein CASP1

|  |  |
| --- | --- |
| URN | urn:agi-llid:834 |
| Total Entities | 0 |
| Connectivity | 4348 |
| Name | CASP1 |
| Description | caspase 1, apoptosis-related cysteine peptidase |
| Notes | This gene encodes a protein which is a member of the cysteine-aspartic acid protease (caspase) family. Sequential activation of caspases plays a central role in the execution-phase of cell apoptosis. Caspases exist as inactive proenzymes which undergo proteolytic processing at conserved aspartic residues to produce 2 subunits, large and small, that dimerize to form the active enzyme. This gene was identified by its ability to proteolytically cleave and activate the inactive precursor of interleukin-1, a cytokine involved in the processes such as inflammation, septic shock, and wound healing. This gene has been shown to induce cell apoptosis and may function in various developmental stages. Studies of a similar gene in mouse suggest a role in the pathogenesis of Huntington disease. Alternative splicing results in transcript variants encoding distinct isoforms. [provided by RefSeq, Mar 2012] |
| ObjectType | Protein |

---

|  |  |
| --- | --- |
| Pathway | NOD-like Receptors |
|  | Dectin-1 (CLEC7A) Signaling |
|  | T-Cell Cytotoxic Mediated Cell Death |
|  | Toll-like Receptor Independent Sterile Inflammation |
|  | Axonal Transport |
|  | Inflammatory Reaction in Acne Vulgaris |
|  | Apoptotic Keratinocytes Clearance Recession in Systemic Lupus Erythematosus |
|  | Dendritic Cell Activation in Systemic Lupus Erythematosis |
|  | Plasmacytoid Dendritic Cell in Diabetes Mellitus Type 1 |
|  | Macrophage Function in Diabetes Mellitus Type 1 |
|  | Conventional Dendritic Cell Role in Diabetes Mellitus Type 1 |
|  | Proteins Involved in non-Alcoholic Fatty Liver Disease |
|  | beta-Cell Destruction in Diabetes Mellitus Type 1 |
|  | Crohn's Disease |
|  | Macrophage Dysfunction |
|  | Proteins Involved in Helicobacter Infections |
|  | Dendritic Cell Function in Ulcerative Colitis |
|  | Epithelial Cell in the Innate Immune Response in Ulcerative Colitis |
|  | Antigen-Presenting Cell Role in Asthma |
|  | Proteins Involved in Gout |
|  | Monocytes Function in Gout |
|  | Proteins Involved in Osteoarthritis |
|  | Dendritic Cell Dysfunction in Psoriatic Arthritis |
|  | PAPA Syndrome |
|  | Mevalonate Kinase Deficiency |
|  | Blau Syndrome |
|  | Cryopyrin (NLPR3) Associated Periodic Syndromes |
|  | Familial Cold Autoinflammatory Syndrome 2 |
|  | Familial Mediterranean Fever |
|  | Majeed Syndrome |
|  | Proteins Involved in Huntington Disease |
|  | Proteins Involved in Retinitis Pigmentosa |
|  | HMGB1 and IL1B in Neuroinflammation |
|  | HMGB1 and IL1B in Neuronal Hyperexcitation |
|  | SOD1 Mutation |
|  | Proteins with Altered Expression in Amyotrophic Lateral Sclerosis |
|  | Proteins Involved in Amyotrophic Lateral Sclerosis |
|  | Proteins Involved in Age-Related Macular Degeneration |
|  | Immune System Role in Otitis Media |
|  | Alveolar Macrophages Dysfunction in COPD |
|  | Asbestosis- and Silicosis-Associated Inflammation Provokes Cancer Development |
|  | Proteins with Altered Expression in Cancer-Associated Resisting to Cell Death |
|  | Genes with Mutations in Cancer-Associated Resisting to Cell Death |
|  | Suppression of Caspase Activity in Cancer |
|  | Apoptosis Evasion in Cancer: Overview |
|  | Elevated Receptors -> Expression Targets in Lymph Node |
|  | TNF Receptors -> Expression Targets in Lymphoid System and Blood |
|  | Elevated Receptors -> Expression Targets in Thymus |
|  | Elevated Receptors -> Expression Targets in Breast |
|  | e |
|  | pathway 4 |
|  | pprarg neg, uknown targets, ps-positive |
|  | prarg neg,ukn expres targets, ps-positive |
|  | PPARG negative targets |
|  | pparg expr target\_regulators |
|  | Neighbors of disease exacerbation |
|  | Neighbors of tumor progression |
|  | Neighbors of inflammatory status |
|  | Neighbors of overall survival |
|  | Neighbors of disease severity |
|  | Neighbors of tumor size |
|  | Neighbors of lesion size |
|  | Neighbors of mortality |
|  | Neighbors of survival rate |
|  | Neighbors of infarct size |
|  | Neighbors of survival time |
|  | Neighbors of insulin sensitivity |
|  | Neighbors of bacterial load |
|  | Neighbors of therapeutic efficacy |
|  | Neighbors of clinical stage |
|  | Neighbors of lethality |
|  | Neighbors of body weight |
|  | Neighbors of body weight gain |
|  | Neighbors of neutrophil count |
|  | Neighbors of glucose tolerance |
|  | Neighbors of morbidity |
|  | Neighbors of mortality rate |
|  | Neighbors of bone mineral density |
|  | Neighbors of radioresistance |
|  | Neighbors of lesion area |
|  | Neighbors of early mortality |
|  | Neighbors of viral load |
|  | Neighbors of muscle mass |
|  | Neighbors of cancer risk |
|  | Neighbors of cancer incidence |
|  | Neighbors of systolic blood pressure |
|  | Neighbors of fasting plasma glucose |
|  | Neighbors of energy expenditure |
|  | Neighbors of left ventricular ejection fraction |
|  | Neighbors of proliferation index |
|  | Neighbors of cell permeability |
|  | Neighbors of gastric cancer risk |
|  | Neighbors of neurological outcome |
|  | Neighbors of infarct area |
|  | Neighbors of recurrence rate |
|  | Neighbors of fractional shortening |
|  | Neighbors of disease exacerbation |
|  | Neighbors of tumor progression |
|  | Neighbors of inflammatory status |
|  | Neighbors of overall survival |
|  | Neighbors of disease severity |
|  | Neighbors of tumor size |
|  | Neighbors of lesion size |
|  | Neighbors of mortality |
|  | Neighbors of survival rate |
|  | Neighbors of infarct size |
|  | Neighbors of survival time |
|  | Neighbors of insulin sensitivity |
|  | Neighbors of bacterial load |
|  | Neighbors of therapeutic efficacy |
|  | Neighbors of clinical stage |
|  | Neighbors of lethality |
|  | Neighbors of body weight |
|  | Neighbors of body weight gain |
|  | Neighbors of neutrophil count |
|  | Neighbors of glucose tolerance |
|  | Neighbors of morbidity |
|  | Neighbors of mortality rate |
|  | Neighbors of bone mineral density |
|  | Neighbors of radioresistance |
|  | Neighbors of lesion area |
|  | Neighbors of early mortality |
|  | Neighbors of viral load |
|  | Neighbors of muscle mass |
|  | Neighbors of cancer risk |
|  | Neighbors of cancer incidence |
|  | Neighbors of systolic blood pressure |
|  | Neighbors of fasting plasma glucose |
|  | Neighbors of energy expenditure |
|  | Neighbors of left ventricular ejection fraction |
|  | Neighbors of proliferation index |
|  | Neighbors of cell permeability |
|  | Neighbors of gastric cancer risk |
|  | Neighbors of neurological outcome |
|  | Neighbors of infarct area |
|  | Neighbors of recurrence rate |
|  | Neighbors of fractional shortening |
|  | Neighbors of cell development |
|  | Neighbors of immune response |
|  | Neighbors of cell population |
|  | Neighbors of T-cell development |
|  | Neighbors of inflammatory response |
|  | Neighbors of wound healing |
|  | Neighbors of angiogenesis |
|  | Neighbors of innate immune response |
|  | Neighbors of cell function |
|  | Neighbors of macrophage activation |
|  | Neighbors of cell formation |
|  | Neighbors of adaptive immune response |
|  | Neighbors of epithelial to mesenchymal transition |
|  | Neighbors of immunity |
|  | Neighbors of chemotaxis |
|  | Neighbors of dendritic cell differentiation |
|  | Neighbors of cell survival |
|  | Neighbors of osteoclast differentiation |
|  | Neighbors of cell phenotype |
|  | Neighbors of cell adhesion |
|  | Neighbors of T-cell activation |
|  | Neighbors of fibrogenesis |
|  | Neighbors of tumor growth |
|  | Neighbors of dendritic cell development |
|  | Neighbors of tissue repair |
|  | Neighbors of cell count |
|  | Neighbors of cell infiltration |
|  | Neighbors of cell interaction |
|  | Neighbors of SMC proliferation |
|  | Neighbors of lumen formation |
|  | Neighbors of adipogenesis |
|  | Neighbors of lipid storage |
|  | Neighbors of cell damage |
|  | Neighbors of aging |
|  | Neighbors of adipocyte differentiation |
|  | Neighbors of macrophage migration |
|  | Neighbors of microglial activation |
|  | Neighbors of neutrophil recruitment |
|  | Neighbors of regeneration |
|  | Neighbors of cell migration |
|  | Neighbors of smooth muscle cell migration |
|  | Neighbors of transendothelial migration |
|  | Neighbors of phagocytosis |
|  | Neighbors of immune system activation |
|  | Neighbors of leukocyte recruitment |
|  | Neighbors of monocyte recruitment |
|  | Neighbors of cell invasion |
|  | Neighbors of heart function |
|  | Neighbors of hemopoiesis |
|  | Neighbors of immune system function |
|  | Neighbors of lung development |
|  | Neighbors of autophagy |
|  | Neighbors of monocyte adhesion |
|  | Neighbors of transcription activation |
|  | Neighbors of T-cell response |
|  | Neighbors of neuroprotection |
|  | Neighbors of stem cell differentiation |
|  | Neighbors of endothelial cell function |
|  | Neighbors of neuronal death |
|  | Neighbors of cell growth |
|  | Neighbors of ROS generation |
|  | Neighbors of epithelial cell proliferation |
|  | Neighbors of sensitization |
|  | Neighbors of macrophage function |
|  | Neighbors of cellular immune response |
|  | Neighbors of macrophage differentiation |
|  | Neighbors of cell differentiation |
|  | Neighbors of life span |
|  | Neighbors of psoriasis |
|  | Neighbors of inflammatory disease |
|  | Neighbors of rheumatoid arthritis |
|  | Neighbors of atherosclerosis |
|  | Neighbors of fibrosis |
|  | Neighbors of colitis |
|  | Neighbors of arthritis |
|  | Neighbors of inflammatory bowel disease |
|  | Neighbors of injury |
|  | Neighbors of atherogenesis |
|  | Neighbors of vascular remodeling |
|  | Neighbors of metastasis |
|  | Neighbors of carcinogenesis |
|  | Neighbors of multiple sclerosis |
|  | Neighbors of chronic inflammation |
|  | Neighbors of inflammation |
|  | Neighbors of obesity |
|  | Neighbors of vascular disease |
|  | Neighbors of diabetes mellitus |
|  | Neighbors of asthma |
|  | Neighbors of pneumonia |
|  | Neighbors of hyperplasia |
|  | Neighbors of osteoarthritis |
|  | Neighbors of liver fibrosis |
|  | Neighbors of chronic obstructive pulmonary disease |
|  | Neighbors of myocardial infarction |
|  | Neighbors of leukocyte infiltration |
|  | Neighbors of reperfusion injury |
|  | Neighbors of airway inflammation |
|  | Neighbors of hepatitis |
|  | Neighbors of insulin resistance |
|  | Neighbors of infection |
|  | Neighbors of vascular injury |
|  | Neighbors of renal injury |
|  | Neighbors of macrophage infiltration |
|  | Neighbors of liver injury |
|  | Neighbors of neuroinflammation |
|  | Neighbors of sepsis |
|  | Neighbors of death |
|  | Neighbors of encephalomyelitis |
|  | Neighbors of autoimmune disease |
|  | Neighbors of ischemic stroke |
|  | Neighbors of dermatitis |
|  | Neighbors of neutrophil infiltration |
|  | Neighbors of heart disease |
|  | Neighbors of diabetic nephropathy |
|  | Neighbors of lung injury |
|  | Neighbors of systemic sclerosis |
|  | Neighbors of hepatocellular carcinoma |
|  | Neighbors of acute lung injury |
|  | Neighbors of gastric cancer |
|  | Neighbors of atherosclerotic plaque |
|  | Neighbors of colorectal cancer |
|  | Neighbors of cardiac remodeling |
|  | Neighbors of ischemia |
|  | Neighbors of systemic lupus erythematosus |
|  | Neighbors of collagen-induced arthritis |
|  | Neighbors of cartilage degeneration |
|  | Neighbors of neoplasm invasion |
|  | Neighbors of kidney disease |
|  | Neighbors of heart failure |
|  | Neighbors of lung disease |
|  | Neighbors of pulmonary fibrosis |
|  | Neighbors of cardiovascular disease |
|  | Neighbors of stroke |
|  | Neighbors of cancer |
|  | Neighbors of cerebral ischemia |
|  | Neighbors of type 2 diabetes |
|  | Neighbors of neoplasm |
|  | Neighbors of neointima |
|  | Neighbors of vasculitis |
|  | Neighbors of atrophy |
|  | Neighbors of diet-induced obesity |
|  | Neighbors of respiratory hypersensitivity |
|  | Neighbors of myocardial fibrosis |
|  | Neighbors of rupture |
|  | Neighbors of endothelial cell dysfunction |
|  | Neighbors of hypertrophy |
|  | Neighbors of hypertension |
|  | Neighbors of intimal hyperplasia |
|  | Neighbors of allograft rejection |
|  | Neighbors of epithelial cell |
|  | Neighbors of keratinocyte |
|  | Neighbors of vascular smooth muscle cell |
|  | Neighbors of monocyte |
|  | Neighbors of macrophage |
|  | Neighbors of adipocyte |
|  | Neighbors of activated monocyte |
|  | Neighbors of fibroblast |
|  | Neighbors of PBMC |
|  | Neighbors of microglia |
|  | Neighbors of mononuclear cell |
|  | Neighbors of dendritic cell |
|  | Neighbors of Kuppfer cell |
|  | Neighbors of proadipocyte |
|  | Neighbors of kidney tubule cell |
|  | Neighbors of bone marrow macrophage |
|  | Neighbors of peritoneal macrophage |
|  | Neighbors of neutrophil |
|  | Neighbors of astrocyte |
|  | Neighbors of bone marrow derived dendritic cell |
|  | Neighbors of mononuclear phagocyte |
|  | Neighbors of immunocompetent cell |
|  | Neighbors of myeloid cell |
|  | Neighbors of stem cell |
|  | Neighbors of sinusoidal cell |
|  | Neighbors of B-cell |
|  | Neighbors of chondrocyte |
|  | Neighbors of T-cell |
|  | Neighbors of neuroglia |
|  | Neighbors of hematopoietic cell |
|  | Neighbors of antigen-presenting cell |
|  | Neighbors of brain cell |
|  | Neighbors of liver |
|  | Neighbors of intestine |
|  | Neighbors of bone |
|  | Neighbors of brain |
|  | Neighbors of artery wall |
|  | Neighbors of NF-kB family |
|  | Neighbors of oxidized LDL |
|  | Neighbors of NFKBI |
|  | Neighbors of PI3K |
|  | Neighbors of STAT family |
|  | Neighbors of mitogen-activated protein kinase |
|  | Neighbors of JAK |
|  | Neighbors of MEK1/2 |
|  | Neighbors of growth factor |
|  | Neighbors of ovalbumin |
|  | Neighbors of chorionic gonadotropin |
|  | Neighbors of LXR |
|  | Neighbors of IL23 |
|  | Neighbors of caspase |
|  | Model of PPARG signaling in psoriasis |
|  | PPARG negative regulators and targets |
|  | sars-cov no binding |
|  | Dendritic Cell Activation in COVID19 |
|  | 1\_Inflammation in keratinocytes in the pathology of acne |
|  | 1\_Insufficient activation of immune response in the middle ear epithelium cells in otitis media |
|  | 3\_Role of alveolar macrophages in pulmonary tissue destruction in COPD |
|  | 1\_Th2 cell response in asthma |
|  | 2\_Paneth cell dysfunction in Crohn's disease |
|  | 1\_Defects in response to pathogens in the gut promotes inflammation in Crohn's disease |
|  | 2\_Intestine epithelial cells drive immunological responses in ulcerative colitis |
|  | 1\_Antigen-presenting cells promote islet dysfunction and immune system activation in T1D |
|  | 3\_Islet beta-cell destruction in T1D |
|  | 2\_Mutations in SOD1 and associated proteins cause motor neuron death in familial ALS |
|  | Figure 4\_Diabetes mellitus Type 1: conventional dendritic cell |
|  | drug-target pairs direct regulation |
|  | drug-target moltransport |

---

|  |  |
| --- | --- |
| Group | other partners for SARS |

---

|  |  |
| --- | --- |
| MedScan ID | 834 |
|  | 12342236 |

---

|  |  |
| --- | --- |
| LocusLink ID | 834 |
|  | 12362 |
|  | 25166 |

---

|  |  |
| --- | --- |
| Alias | interleukin 1beta precursor proteinase |
|  | caspase 1 isoform gamma |
|  | Interleukin-1 converting enzyme |
|  | pro-interleukin 1beta proteinase |
|  | pro-interleukin 1beta proteinases |
|  | pro-interleukin 1beta protease |
|  | prointerleukin 1beta proteases |
|  | prointerleukin 1beta proteinase |
|  | Casp I |
|  | IL1BC |
|  | interleukin 1, beta, convertase |
|  | apoptosis-related cysteine proteinase caspase 1 |
|  | pro-interleukin 1beta proteases |
|  | caspase 1 |
|  | interleukin-1beta convertases |
|  | prointerleukin 1beta proteinases |
|  | interleukin 1beta precursor proteases |
|  | caspase 1 isoform beta |
|  | interleukin IL1BC |
|  | interleukin 1beta precursor proteinases |
|  | interleukin 1-beta converting enzymes |
|  | IL-1B converting enzyme |
|  | caspase 1, apoptosis-related cysteine peptidase (interleukin 1, beta, convertase) |
|  | caspase 1, apoptosis-related cysteine proteinase (interleukin 1, beta, convertase) |
|  | IL1 converting enzyme |
|  | interleukin 1-B converting enzyme |
|  | ICEs |
|  | IL-1 beta converting enzyme |
|  | interleukin 1 beta-converting enzyme |
|  | interleukin IL1B-convertase |
|  | Interleukin IL1 converting enzyme |
|  | caspase I |
|  | caspase 1 isoform epsilon |
|  | caspase 1 isoform delta |
|  | caspase 1, apoptosis-related cysteine protease (interleukin 1, beta, convertase) |
|  | IL1BCE |
|  | CASP1 nirs variant 1 |
|  | apoptosis-related cysteine protease caspase 1 |
|  | 3.4.22.36 |
|  | apoptosis-related cysteine peptidase caspase 1 |
|  | interleukin IL1-beta convertase |
|  | interleukin IL-1 beta converting enzyme |
|  | interleukin IL1beta-converting endopeptidase |
|  | interleukin 1beta precursor protease |
|  | caspase 1, apoptosis-related cysteine peptidase |
|  | I1BC |
|  | interleukin IL1-B converting enzyme |
|  | ICE |
|  | interleukin 1 beta-converting endopeptidase |
|  | IL1B-convertase |
|  | prointerleukin 1beta protease |
|  | I1BC\_HUMAN |
|  | IL1beta-converting endopeptidase |
|  | interleukin IL1BCE |
|  | CASP1 |
|  | interleukin 1beta-converting endopeptidases |
|  | caspase 1 isoform alpha |
|  | P45 |
|  | IL-1 beta-converting enzyme |
|  | caspase-1 |
|  | CASP-1 |
|  | IL-1BC |
|  | interleukin-1 beta convertase |
|  | interleukin-1 beta-converting enzyme |
|  | Interleukin 1beta converting enzyme |
|  | interleukin 1-beta converting enzyme |
|  | interleukin 1-beta-converting endopeptidase |
|  | interleukin-1 beta convertase precursor |
|  | interleukin 1 converting enzyme |
|  | interleukin 1-beta convertase |

---

|  |  |
| --- | --- |
| EC Number | 3.4.22.36 |

---

|  |  |
| --- | --- |
| GO ID | 0050700 |
|  | 0008656 |
|  | 0004197 |
|  | 0097153 |
|  | 0097199 |
|  | 0004175 |
|  | 0019900 |
|  | 0006919 |
|  | 0006915 |
|  | 0097190 |
|  | 0071345 |
|  | 0071346 |
|  | 0071222 |
|  | 0071260 |
|  | 0071310 |
|  | 0019221 |
|  | 0097194 |
|  | 0043123 |
|  | 0043280 |
|  | 0032731 |
|  | 0050718 |
|  | 1903265 |
|  | 0016540 |
|  | 0006508 |
|  | 0042981 |
|  | 0007165 |
|  | 0097169 |
|  | 0072557 |
|  | 0072558 |
|  | 0072559 |
|  | 0005737 |
|  | 0005829 |
|  | 0005886 |
|  | 0097179 |
|  | 0032991 |
|  | 0008234 |
|  | 0016787 |
|  | 0008233 |
|  | 0097110 |
|  | 0032611 |
|  | 0060081 |
|  | 0007613 |
|  | 0001774 |
|  | 0051882 |
|  | 0007520 |
|  | 0043065 |
|  | 0046010 |
|  | 0050715 |
|  | 0050717 |
|  | 0097300 |
|  | 0016485 |
|  | 0070269 |
|  | 0010506 |
|  | 0050727 |
|  | 0033198 |
|  | 0009617 |
|  | 0042493 |
|  | 0001666 |
|  | 0032496 |
|  | 0014070 |
|  | 1901998 |
|  | 0005576 |
|  | 0043005 |
|  | 0005634 |
|  | 0030324 |
|  | 0007494 |
|  | 0009636 |
|  | 0005739 |
|  | 0005622 |
|  | 0008219 |
|  | 0097200 |
|  | 0045087 |
|  | 0006917 |
|  | 0035872 |
|  | 0005515 |
|  | 0042221 |
|  | 0043234 |
|  | 0008152 |

---

|  |  |
| --- | --- |
| KEGG ID | hsa:834 |
|  | mmu:12362 |
|  | rno:25166 |

---

|  |  |
| --- | --- |
| Organism | Homo sapiens {Organism urn:agi-taxid:9606} |
|  | Mus musculus {Organism urn:agi-taxid:10090} |
|  | Rattus norvegicus {Organism urn:agi-taxid:10116} |
|  | Homo sapiens |
|  | Mus musculus |
|  | Rattus norvegicus |

---

|  |  |
| --- | --- |
| Mouse chromosome position | 9 2.46 cM |
|  | 9 1.0 cM |

---

|  |  |
| --- | --- |
| OMIM ID | 147678 |

---

|  |  |
| --- | --- |
| Rat chromosome position | 8q11 |

---

|  |  |
| --- | --- |
| Hugo ID | 1499 |
|  | HGNC:1499 |

---

|  |  |
| --- | --- |
| Human chromosome position | 11q22.3 |
|  | 11q23 |

---

|  |  |
| --- | --- |
| Swiss-Prot Accession | P29466 |
|  | A8K249 |
|  | A8K257 |
|  | P29466.1 |
|  | P29452 |
|  | P29452.1 |
|  | Q91W32 |
|  | P43527.1 |
|  | B5MDZ1 |
|  | Q53EY6 |
|  | Q6DMQ1 |
|  | Q6GSS3 |
|  | Q6PI75 |
|  | Q9UCN3 |
|  | P43527 |
|  | Q5FBZ0 |

---

|  |  |
| --- | --- |
| PIR ID | A54263 |
|  | A56084 |
|  | B56084 |
|  | C56084 |
|  | D56084 |
|  | A46495 |

---

|  |  |
| --- | --- |
| GenBank ID | NC\_000011 |
|  | NM\_033293 |
|  | NP\_150635 |
|  | NM\_001223 |
|  | NP\_001214 |
|  | NM\_033292 |
|  | NP\_150634 |
|  | XM\_017018395 |
|  | XP\_016873884 |
|  | XM\_017018394 |
|  | XP\_016873883 |
|  | XM\_017018393 |
|  | XP\_016873882 |
|  | NM\_033295 |
|  | NP\_150637 |
|  | NM\_033294 |
|  | NP\_150636 |
|  | NM\_001257119 |
|  | NP\_001244048 |
|  | NM\_001257118 |
|  | NP\_001244047 |
|  | XM\_017018396 |
|  | XP\_016873885 |
|  | NG\_029124 |
|  | AP001153 |
|  | CH471065 |
|  | EAW67057 |
|  | EAW67058 |
|  | EAW67059 |
|  | EAW67060 |
|  | EAW67061 |
|  | KF455483 |
|  | L27475 |
|  | AAD15243 |
|  | AA505065 |
|  | AA737419 |
|  | AA761907 |
|  | AB102878 |
|  | BAD89407 |
|  | AK223503 |
|  | BAD97223 |
|  | AK290114 |
|  | BAF82803 |
|  | AK290122 |
|  | BAF82811 |
|  | AK296646 |
|  | BAG59246 |
|  | AK301037 |
|  | BAG62650 |
|  | AK310059 |
|  | AK313516 |
|  | BAG36296 |
|  | AY660536 |
|  | AAT72297 |
|  | BC041689 |
|  | AAH41689 |
|  | BC062327 |
|  | AAH62327 |
|  | M87507 |
|  | AAA66942 |
|  | U13697 |
|  | AAC50107 |
|  | U13698 |
|  | AAC50108 |
|  | U13699 |
|  | AAC50109 |
|  | U13700 |
|  | AAC50110 |
|  | X65019 |
|  | CAA46153 |
|  | P29466 |
|  | NC\_000075 |
|  | NM\_009807 |
|  | NP\_033937 |
|  | AC141637 |
|  | CH466636 |
|  | EDL09320 |
|  | EDL09321 |
|  | EDL09322 |
|  | EDL09323 |
|  | U04269 |
|  | AAA56992 |
|  | AK132826 |
|  | AK163069 |
|  | BC008152 |
|  | AAH08152 |
|  | L03799 |
|  | AAA39306 |
|  | L28095 |
|  | AAA20209 |
|  | P29452 |
|  | NC\_005107 |
|  | NM\_012762 |
|  | NP\_036894 |
|  | AC\_000076 |
|  | AABR07068939 |
|  | AAHX01052962 |
|  | CH474089 |
|  | EDL85232 |
|  | EDL85233 |
|  | BC088098 |
|  | D85899 |
|  | BAB67770 |
|  | FQ228818 |
|  | S79676 |
|  | AAB35431 |
|  | U14647 |
|  | AAA85812 |
|  | U34621 |
|  | AAC52259 |
|  | P43527 |
|  | AABR06052953 |
|  | AAHY01030335 |
|  | HQ447656 |
|  | ABBA01039912 |
|  | AC\_000025 |
|  | XM\_011543017 |
|  | XM\_011543018 |
|  | ADQ32141 |
|  | XP\_011541319 |
|  | AC\_000143 |
|  | NC\_018922 |
|  | XM\_006718924 |
|  | XP\_006718987 |
|  | XP\_011541320 |
|  | AMYH02025506 |
|  | NT\_033899 |
|  | NW\_001838042 |
|  | NW\_925173 |
|  | AC\_000054 |
|  | CR592117 |
|  | Q5FBZ0 |
|  | NT\_039471 |
|  | NW\_001030736 |
|  | NW\_047797 |
|  | NW\_001084863 |
|  | Q91W32 |

---

|  |  |
| --- | --- |
| Swiss-Prot ID | CASP1\_HUMAN |
|  | CASP1\_MOUSE |
|  | Q91W32\_RAT |

---

|  |  |
| --- | --- |
| Cell Localization | Cytoplasm |
|  | Cell membrane |

---

|  |  |
| --- | --- |
| Ensembl ID | ENSG00000137752 |
|  | ENSP00000432340.1 |
|  | ENST00000527979.5 |
|  | ENSP00000433138.1 |
|  | ENST00000533400.6 |
|  | ENSP00000434779.1 |
|  | ENST00000525825.5 |
|  | ENSP00000410076.3 |
|  | ENST00000436863.7 |
|  | ENSP00000434250.1 |
|  | ENST00000526568.5 |
|  | ENSP00000403260.1 |
|  | ENST00000446369.5 |
|  | ENSP00000344132.5 |
|  | ENST00000353247.9 |
|  | ENSMUSG00000025888 |
|  | ENSMUSP00000027015.5 |
|  | ENSMUST00000027015.5 |
|  | ENSRNOG00000007372 |
|  | ENSRNOP00000009993.5 |
|  | ENSRNOT00000009993.6 |
|  | ENST00000525825 |
|  | ENST00000353247 |
|  | ENSRNOP00000009993 |
|  | ENST00000393136 |
|  | ENSP00000432340 |
|  | ENSMUST00000027015 |
|  | ENST00000533400 |
|  | ENST00000533400.5 |
|  | ENSP00000433138 |
|  | ENST00000436863 |
|  | ENSP00000410076 |
|  | ENST00000527979 |
|  | ENST00000526568 |
|  | ENSMUSP00000027015 |
|  | ENSRNOT00000009993 |
|  | ENST00000446369 |
|  | ENSP00000376844 |
|  | ENSP00000403260 |
|  | ENSP00000434779 |
|  | ENSP00000344132 |
|  | ENSP00000434250 |

---

|  |  |
| --- | --- |
| MGI ID | MGI:96544 |
|  | 96544 |

---

|  |  |
| --- | --- |
| RGD ID | 2274 |

---

|  |  |
| --- | --- |
| Unigene ID | Hs.2490 |
|  | Rn.37508 |
|  | Mm.1051 |

---

|  |  |
| --- | --- |
| Homologene ID | 7653 |
|  | 133272 |

---

|  |  |
| --- | --- |
| IPI ID | IPI00219307 |
|  | IPI00219308 |
|  | IPI00219309 |
|  | IPI00021800 |
|  | IPI00219310 |
|  | IPI00125676 |
|  | IPI00201423 |
|  | IPI00797075 |
|  | IPI00903240 |
|  | IPI00554551 |

---
